# 

**Published:** 1903-03

**Authors:** 


*Vol. XXI.* *No. 79.*

NewYork

### THE BRISTOL MEDICO-CHIRURGICAL JOURNAL

*PUBLISHED UNDER THE AUSPICES OF*

*THE BRISTOL MEDICO-CHIRURGICAL SOCIETY.*

*Editor:* R. SHINGLETON SMITH, M.D., B.Sc.

WITH WHOM ARE ASSOCIATED

J. MICHELL CLARKE, M.A., M.D.,
J. LACY FIRTH, M.S., M.D., JAMES SWAIN, M.S., M.D.
P. WATSON WILLIAMS, M.D., *Assistant Editor.*
*Editorial Secretary:* JAMES TAYLOR.

| | |
|---|---|
| *Bath* ... ... EDWARD J. CAVE, M.D. | *Newport* ... A. GARROD THOMAS, M.D., C.M. |
| *Cardiff* ... ... D. R. PATERSON, M.D., C.M. | *Plymouth* . PAUL SWAIN, F.R.C.S. |
| *Cheltenham* ... E. T. WILSON, M.B. | *Swansea* ... E. LECRONIER LANCASTER, B.A., M.B., B.Ch. |
| *Exeter* ... ... W. GORDON, M.A., M.D. | *Swindon* ... J. CAMPBELL MACLEAN, M.B., C.M. |
| *Gloucester* ... RAYNER W. BATTEN, M.D. | *Weston-s.-Mare* R. ROXBURGH, M.B. |

BRISTOL: J. W. ARROWSMITH.

LONDON: J. & A. CHURCHILL, 7 GREAT MARLBOROUGH STREET.

*Price One Shilling and Sixpence.*

